# Risk factors for recurrence of pelvic organ prolapse after vaginal surgery among Ugandan women: a prospective cohort study

**DOI:** 10.1007/s00192-021-04930-8

**Published:** 2021-07-28

**Authors:** Musa Kayondo, Verena Geissbüehler, Richard Migisha, Rogers Kajabwangu, Joseph Njagi, Paul Kalyebara Kato, Yarine Fajardo, Henry Mark Lugobe, Dan Kabonge Kaye

**Affiliations:** 1 Faculty of Medicine, Mbarara University of Science and Technology, P.O.BOX 1410, Mbarara, Uganda; 2 Department of Obstetrics and Gynecology, Mbarara Regional Referral Hospital, P.O.BOX 40, Mbarara, Uganda; 3 Department of Gynecology, St. Claraspital, Basel, Switzerland; 4 Department of Physiology, Faculty of Medicine, Mbarara University of Science and Technology, P.O.BOX 1410, Mbarara, Uganda; 5 Department of Obstetrics and Gynecology, Makerere University College of Health Sciences, Kampala, Uganda

**Keywords:** Pelvic organ prolapse, Prolapse recurrence, Risk factors, Surgery

## Abstract

**Introduction and hypothesis:**

This study was aimed at determining the recurrence rate and risk factors for the recurrence of pelvic organ prolapse (POP), at 1 year post-vaginal reconstructive surgery in a resource-limited setting.

**Methods:**

We enrolled women who underwent vaginal surgery for POP at the urogynecology unit of Mbarara Regional Referral Hospital (MRRH) in southwestern Uganda between December 2018 and February 2020. The surgeries that were performed include anterior colporrhaphy for cystocele, posterior colporrhaphy for rectocele, vaginal hysterectomy with vault suspension for uterine prolapse, and cervicopexy in those with uterine prolapse where uterine-sparing surgery was desired. The women were followed up for a period of 1 year after surgery. Pelvic examinations in lithotomy position under maximum strain were carried out to assess for recurrence using the Pelvic Organ Quantification (POP-Q) system. Recurrence was defined as a prolapse of ≥POP-Q stage II. Descriptive analyses and multivariate log binomial regression were performed to determine risk factors for recurrence.

**Results:**

Of the 140 participants enrolled, 127 (90.7%) completed the follow-up at 1 year. The recurrence rate was 25.2% (32 out of 127). Most (56.3%) of the recurrences occurred in the anterior compartment and in the same site previously operated. Women aged <60 years (RR = 2.34; 95% CI: 1.16–4.72; *p* = 0.018) and those who had postoperative vaginal cuff infection (RR = 2.54; 95% CI: 1.5–4.3; *p* = 0.001) were at risk of recurrence.

**Conclusion:**

Recurrence of POP was common. Younger women, and those with postoperative vaginal cuff infection, were more likely to experience recurrent prolapse after vaginal repair.

## Introduction

Pelvic organ prolapse (POP) is defined as the descent of one or more aspects of the vagina or uterus. This descent may be of the anterior vaginal wall, posterior vaginal wall, uterus, vaginal vault after hysterectomy, or a combination of these, which is as a result of failure of their support mechanisms [1]. There are limited data on the prevalence of POP in sub-Saharan Africa (SSA) including Uganda. However, high rates of 23.5% have been reported in Ethiopia [2]. POP affects the quality of life of women [3], hence requiring management. One of the main modes of management of symptomatic POP is surgery. Conservative methods such as pessaries have also been shown to be effective in the management of POP as an alternative to surgery [4, 5]. However, pessaries are not readily available; therefore, their use among clinicians in the treatment of symptomatic POP in our setting is low [6].

The lifetime risk of undergoing surgery to correct POP is 11.9% [7, 8] and close to 200,000 women undergo surgery for POP in the USA annually [9, 10]. Surgery for POP has a number of adverse outcomes, among them being recurrence [11]. The recurrence rates seem to differ from one study to another. Whiteside et al. [12] reported a recurrence rate of 58% after 1 year of follow-up, whereas in studies where the follow-up period was 5 years, recurrence was between 13 and 31% [13, 14]. Another study found a recurrence rate of 25% after a 10-year retrospective follow-up [15].

Several factors have been found to influence recurrence of POP; among them, age < 60 years, postmenopausal status, advanced stages of POP (III–IV), history of complicated delivery, urinary incontinence before POP surgery, widened genital hiatus, high body mass index (BMI), and failure to perform apical suspension [12, 13, 16, 17]. In various studies, some women in whom POP recurred after surgery often underwent repeat surgery, with reported reoperation rates ranging between 10 and 17% [13, 18]. Although recurrence does not necessarily translate into reoperation, these repeat surgeries, however small in number, are still a burden on the health system in resource-limited countries where supplies and surgeons required for these repeat operations are not readily available [19].

There is a paucity of data on the recurrence of POP after surgery and factors associated with such recurrence in sub-Saharan Africa; yet, this is crucial to inform evidence-based interventions aimed at reducing the risk of recurrence, as well as the risk of reoperation in resource-limited settings. Therefore, in this study, we aimed to determine the recurrence rate of, and risk factors for the recurrence of POP at 1 year after vaginal surgery at a tertiary referral hospital, Mbarara, in rural Southwestern Uganda, to fill this knowledge gap.

## Materials and methods

### Study setting

We conducted the study at the Urogynecology unit of Mbarara Regional Referral Hospital (MRRH) from 1 December 2018 to 1 March 2021. MRRH is a tertiary hospital located in Mbarara district in Southwestern Uganda, about 250 km from the capital city of Kampala. MRRH is the main referral hospital of the entire southwestern Uganda area, serving over 10 districts, and also receives patients from the neighboring countries of Tanzania, Rwanda, Burundi, and the Eastern Democratic Republic of Congo (DRC).

### Study design

This was a prospective cohort study of women with POP who underwent vaginal surgery for POP and were followed up to 1 year post-surgery. Characteristics of women who developed recurrence of POP within 1 year of surgery were compared with those who had not developed recurrence.

### Study population

We enrolled women diagnosed with symptomatic POP who underwent vaginal surgery for POP at MRRH and consented to take part in the study. Participants were considered to have POP if they had any one of the following clinical diagnoses: cystocele, urethrocele, cystourethrocele, uterine prolapse, vault prolapse, enterocele, or rectocele. We excluded women who had had previous POP surgery and those in whom the prolapse operation was performed through the abdominal route. Categorization and staging of POP were done using the Pelvic Organ Prolapse Quantification (POP-Q) system validated by the International Continence Society (ICS) into stages I, II, III, and IV [20, 21], with the woman in lithotomy position under maximal strain [22]. Those eligible for surgery were participants with POP-Q stages II, III, and IV. Surgical eligibility was evaluated by the clinical care team.

### Surgery

The participants underwent surgery for the management of POP after obtaining informed consent. The surgeries were performed for a period of 15 months between December 2018 and February 2020. Surgery was dependent on the type of prolapse. The different types of surgeries that were performed include anterior colporrhaphy for cystocele, posterior colporrhaphy for rectocele, and vaginal hysterectomy with vault suspension (sacrospinous ligament or uterosacral vault suspension) for uterine prolapse in those who had completed child bearing and did not desire uterine-sparing surgery. Cervicopexy was performed in those with uterine prolapse who had not completed child bearing or wanted uterine-sparing surgery. In some women, a combination of procedures was performed to correct the prolapse. All surgeries were performed by a team of subspecialty surgeons (certified urogynecologists), as part of the routine management of POP at the hospital.

### Data collection

A data capture tool was used to collect information on the baseline characteristics of the study participants, intraoperative findings and the postoperative follow-up information. The baseline characteristics included:

1. Sociodemographic characteristics (age, marital status, and smoking
2. Medical history (chronic cough and chronic diseases such as HIV)
3. Gynecological history (parity, menopausal status and history of gynecological operation)
4. Primary POP description (type of prolapse: anterior vaginal wall, posterior wall, uterine, vault prolapse, and preoperative POP-Q stage)

The intraoperative information that was collected included:

1. Surgical procedure performed
2. length of surgery in minutes
3. Intraoperative complications (injury to the bladder, bowel or ureters, and hemorrhage that required transfusion)

Postoperative information collected included:

1. Postoperative complications (vaginal cuff bleeding requiring intervention and vaginal cuff infection)
2. Days spent on the ward after surgery

A participant was judged to have vaginal cuff infection if she had increasing lower abdominal pain, purulent vaginal discharge, and a tender surgical site on physical examination [23]. The data capture form was filled out by the trained research assistants (nurses and surgeons).

### Follow-up of the participants

After discharge from hospital, the participants were followed up for 1 year after surgery to assess for recurrence of POP. Participants were contacted through a phone call 1 week prior to their scheduled visit. The purpose of the call was to remind the participants of their scheduled follow-up visit. Participants who could not be reached on the phone were traced using the contact of their next of kin. This was to minimize loss to follow-up. At each follow-up visit, a pelvic examination with the participant in lithotomy position under maximal strain was carried out to assess for recurrence. This assessment was performed by a trained research assistant (a gynecologist) who was not part of the initial surgical team. A participant was considered to have recurrence if she had a bulge ≥POP-Q stage II on maximum straining [12]. The recurrences were also described according to the location (anterior, posterior, apical compartment) and nature (new site or same site recurrence). In addition, we assessed the women for symptoms of recurrence by asking them whether they felt a bothersome vaginal bulge similar to that before surgery.

### Statistical analysis

Data were entered into Redcap and exported to Stata 13 (StataCorp, College Station, TX, USA) for analysis. Categorical data were presented as frequencies. The recurrence rate was determined by dividing the number of women who had recurrence of POP at 1 year by the total number of women who came for follow-up visits and expressed as a percentage. Differences in demographic and clinical characteristics comparing those with recurrence of POP and those without recurrence were assessed using Chi-squared test or Fisher’s exact test. To compare continuous variables, Wilcoxon rank-sum test was used for non-normally distributed continuous variables (duration of hospital stay) and Student’s *t* tests for normally distributed continuous variables (age, duration of surgery).

To determine the risk factors of recurrence of POP, univariate and multivariate analysis were performed using log binomial regression analysis. Risk ratios (RRs) and their corresponding 95% confidence intervals (CIs) were reported as the measures of association. Factors with *p* value <0.2 at univariate analysis were included in the final multivariate model to determine the adjusted risk factors for recurrence of POP. Additionally, pre-surgery POP-Q stage and parity were included in the final multivariate model because of their known interactive influence on the recurrence of POP after surgery. A *p* value <0.05 was considered statistically significant.

### Ethical considerations

Ethical approvals were obtained from the Mbarara University of Science and Technology (MUST) Research Ethics Committee and the Uganda National Council for Science and Technology (UNCST) number HS368ES. We informed the participants of the study objectives and only those who gave written consent were recruited. Confidentiality was observed during all the interviews and examinations. The participants were assigned study identification numbers.

## Results

A total of 140 women were enrolled. Of these, 127 completed the follow-up period of 1 year and 13 were lost to follow-up giving a completion rate of 90.7%. Therefore, the results being reported are for 127 participants. Of the 127 women who completed the follow-up period, the cumulative number of women with recurrence was 32. The recurrence rate of POP at 1 year after vaginal surgery was 25.2% (95%CI: 17.6–32.8%).

The baseline participant characteristics are shown in Table 1. The mean age of the participants was 55 years (SD ± 15). The majority were of parity >3 (*n* = 109, 85.8%) and postmenopausal (*n* = 82, 64.6%). The majority of the participants had a preoperative POP-Q stage > II (*n* = 100, 78.7%). All the participants received preoperative antibiotics, and all operations were performed under spinal anesthesia. The commonest surgery performed was a transvaginal hysterectomy (TVH) combined with anterior repair plus vault fixation (*n* = 55, 43.3%). Surgery that involved a combination of two or more procedures was performed in the majority of the women (*n* = 88, 69.3%). Intraoperative complications were encountered in 4 patients and these included: 2 rectal injuries, 1 hemorrhage that required blood transfusion, and 1 urinary bladder injury. The main postoperative complication was vaginal cuff infection, which occurred in 10 (7.8%) of the participants.

**Table 1:** Demographic, clinical, and perioperative characteristics of the study participants by recurrence status

Characteristic	Total cohort (*N* = 127)	Recurrence		
		Yes (*n* = 32)	No (*n* = 95)	*p* value
Age in years, mean (SD)	55 (±15)	51 (±13)	57 (±15)	0.054
History of smoking, *n* (%)	40 (31.5)	7 (21.9)	33 (34.7)	0.176
HIV positive, *n* (%)	13 (10.2)	2 (6.3)	11 (11.6)	0.514
History of chronic cough, *n* (%)	9 (7.1)	2 (6.3)	7 (7.4)	0.831
Parity, *n* (%)				0.391
0–3	18 (14.2)	6 (18.8)	12 (12.6)	
4 and above	109 (85.8)	26 (81.3)	83 (87.4)	
Reached menopause, *n* (%)	82 (64.6)	21 (65.6)	61 (64.2)	0.885
History of gynecological operation, *n* (%)	14 (11.0)	4 (12.5)	10 (10.5)	0.758
Site of prolapse, *n* (%)				
Anterior vaginal wall	94 (74.0)	20 (62.5)	74 (77.9)	0.086
Posterior vaginal wall	37 (29.1)	8 (25.0)	29 (30.5)	0.552
Uterine prolapse	92 (72.4)	23 (71.9)	69 (72.6)	0.934
Vault prolapse	6 (4.7)	2 (6.3)	4 (4.2)	0.641
Enterocele	12 (9.5)	2 (6.3)	10 (10.5)	0.729
Preoperative POP-Q stage, *n* (%)				0.638
Stage II	27 (21.3)	7 (21.9)	20 (21.1)	
Stage III	61 (48.0)	15 (46.9)	46 (48.4)	
Stage IV	39 (30.7)	10 (31.3)	29 (30.5)	
Prolapse surgery type, *n* (%)				0.057
Anterior repair alone	12 (9.5)	3 (9.4)	9 (9.5)	
Posterior repair alone	15 (11.8)	3 (9.4)	12 (12.6)	
Sacrospinous cervicopexy	5 (3.9)	3 (9.4)	2 (2.1)	
TVH with anterior and posterior repair plus vault fixation	16 (12.6)	3 (9.4)	13 (13.7)	
TVH with anterior repair plus vault fixation	55 (43.3)	9 (28.1)	46 (48.4)	
TVH alone with vault fixation	17 (13.4)	8 (25.0)	9 (9.5)	
Vault fixation alone for vault prolapse	3 (2.4)	2 (6.3)	1 (1.1)	
Type of vault fixation, *n* (%)				0.343
Sacrospinous	67 (52.8)	20 (62.5)	47 (49.5)	
Uterosacral	31 (24.4)	5 (15.6)	26 (27.4)	
Duration of operation in minutes, mean (SD)	81 (±30)	77 (±34)	83 (±28)	0.3374
Days of hospitalization, median (IQR)	5 (4–6)	5 (4–5)	4 (4–5)	0.2078
Postoperative vaginal cuff infection, *n* (%)	10 (7.9)	4 (12.5)	6 (6.3)	0.27

*IQR* inter-quartile range, *POP-Q* pelvic organ prolapse quantification, *SD* standard deviation, *TVH* total vaginal hysterectomy

The description of recurrence with regard to site and nature is shown in Table 2. Most of the recurrences (bulge of POPQ stage ≥ II) occurred in the anterior compartment (*n* = 18, 56.3%). Eighteen women (56.3%) experienced a recurrence in the same site that had been operated on. Of the 14 new site recurrences, the majority (*n* = 9, 64%) occurred in the anterior compartment. Of the 32 women with recurrence, the majority (68.8%) were asymptomatic. Repeat surgery was performed in only 3 women (9%), whereas the majority (29, 91%) declined to have any form of management, despite the fact that a proportion of these (7 out of 29) were symptomatic.

**Table 2:** Description of recurrent POP among 32 women with recurrence

Characteristic of recurrence		Total with recurrence, *N* = 32	
		Frequency	Percentage
Type of recurrence	Anterior vaginal wall prolapse	18	56.3
	Posterior vaginal wall prolapse	4	12.5
	Uterine prolapse	6	18.8
	Vault prolapse	4	12.5
Nature of recurrence	New site	14	43.8
	Same site	18	56.3
Management of recurrent POP	Repeat surgery	3	9.0
	None	29	91.0
Symptomatic recurrence	Yes	10	31.2
	No	22	68.8

In the adjusted analysis, we found that women aged <60 years (RR = 2.34; 95%CI: 1.16–4.72; *p* = 0.018) and women who had postoperative vaginal cuff infection (RR 2.5; 95% CI, 1.5–4.3; *p* = 0.001) were at risk of recurrence of POP 1 year after surgery as shown in Table 3.

**Table 3:** Risk factors for recurrence of POP

Characteristic	Percentage recurrence (*n* = 32)	Univariate analysis		Multivariate analysis	
	*n*/*N* (%)	RR (95% CI)	*p* value	Adjusted RR (95% CI)	*p* value
Age category					
≥60 years	7/49 (14.3)	Ref		Ref	
<60 years	25/78 (32.1)	2.12 (1.05–4.28)	0.037	2.34 (1.16–4.72)	0.018
Parity					
0–3	6/18 (33.3)	Ref		Ref	
4 & above	26/109 (23.9)	0.72 (0.34–1.49)	0.372	1.06 (0.53–2.12)	0.870
Pre-surgery POPQ stage					
Stage II	7/27(25.9)	Ref		Ref	
Stage III – IV	25/100 (25.0)	0.96 (0.46–1.99)	0.921	1.01 (0.54–1.91)	0.968
Anterior vaginal wall prolapse					
No	12/33 (36.4)	Ref		Ref	
Yes	20/94 (21.3)	0.59 (0.32–1.06)	0.078	0.64 (0.37–1.13)	0.125
Postoperative vaginal cuff infection					
No	28/117 (23.9)	Ref		Ref	
Yes	4/10 (40.0)	1.67 (0.36–3.81)	0.222	2.54 (1.50–4.30)	0.001

*CI* confidence interval, *RR* risk ratio, *Ref* reference category

## Discussion

This hospital-based prospective study determined the recurrence rate of POP at 1 year after surgery and risk factors for the recurrence among women seeking care for POP at MRRH in southwestern Uganda. We found a recurrence rate of 25.2% at 1 year after surgery. Risk factors for recurrence were age (<60 years) and postoperative vaginal cuff infection.

The recurrence rate in this study is comparable with that of other studies carried out in South Korea and Finland, with follow-up periods of up to 2 years, that reported recurrence rates of 17 and 21% respectively [24, 25]. However, another study by Vakili et al. [16] reported a higher recurrence rate of 58% at 1 year after surgery. This contrast could be due to differences in the definitions of recurrence used. Vakili et al. [16] defined recurrence as any prolapse beyond stage zero according to the POP-Q system, whereas in this study we used POP-Q stage II or greater to define recurrence, as in other studies [12, 13, 15, 24].

In agreement with previous studies, most of the recurrences occurred in the anterior compartment and were at the same site that had been operated on before [12, 13, 15, 16]. Some authors reported anterior compartment recurrence rates of up to 8% after POP surgery with vault fixation [26]; however, it has was shown by Eilber et al. [17] that vault suspension is normally protective against recurrence of anterior compartment prolapse. This therefore suggests that recurrences in the anterior compartment are not entirely due to surgical failure but may reflect weak endopelvic tissue in that compartment [27]. The recurrence at the same site may be because the primary surgery to correct the anatomical defect may not have taken care of the underlying neuromuscular and connective tissue dysfunction that was responsible for the primary prolapse and this could have played a significant role in the recurrence [8, 28]. Recurrences (43%) were also observed in a new compartment. This is similar to a study by Price et al. [29] in which the recurrence in a new compartment was 61.5%. This could be explained by the concept of redistribution of forces to other compartments after the primary operation, which predisposes them to prolapse [7, 12, 14].

In our study, women aged <60 years and women with postoperative vaginal cuff infection were at risk of recurrence. The age factor is in correlation with other studies [12, 13, 25]. This may be because younger women may have more inherent pathophysiological pelvic floor issues such as poor tissue quality and more neuromusculo-fascial damage compared with older women with the same degree of prolapse [12, 25]. Furthermore, young women in our low-resource settings continue to engage in physically strenuous work even after surgery in order to earn a living, unlike the older women whose activity may be limited by underlying medical conditions [30]. Postoperative vaginal cuff infection was also found to be a risk factor for recurrence of POP in this study. Nieminen et al. [25] also found postoperative cuff infection to be the most important risk factor for recurrence. Infection in the vaginal cuff may lead to weakening of the fascial tissues leading to displacement of the sutures, resulting in recurrence [18].

In contrast to previous studies [9, 13], advanced stage of prolapse was not found to be a risk factor for recurrence. Similarly, Fialkow et al. [15] did not find any association between advanced stage of POP and recurrence. The reason for this could be that in these two studies, most of the participants with advanced POP underwent a combination of procedures to correct the prolapse during surgery.

Our study had some limitations: it was performed in a single tertiary facility and therefore generalizability should be restricted in a similar manner to those undergoing surgery for POP in other peri-urban sub-Saharan African settings. Genital hiatus, pelvic floor strength, and BMI are not routinely measured in patients in our setting and therefore were not studied. We did not assess symptoms of recurrence using validated pelvic floor dysfunction questionnaires, which could have underestimated the presence of symptoms in our study population. Furthermore, we did not determine whether the duration and complexity of surgery were associated with vaginal cuff infection. Therefore, it was difficult to determine whether longer or difficult surgeries were the reasons for the cuff infections leading to recurrence. Finally, it was also not possible to perform a stratified analysis to determine the risk factors of recurrence for the different types of surgeries because we performed a heterogeneous mixture of procedures in this study and therefore we could not link recurrence to a particular procedure.

## Conclusions

Recurrence of POP after surgery in our setting was common, being detected in about one-quarter of women. Younger women aged <60 years and those who suffered postoperative vaginal cuff infection had an increased risk of recurrence of POP. We recommend that surgeons should put in place measures that minimize the risk of postoperative cuff infection in order to reduce the risk of POP recurrence. Conservative means of treatment for POP such as pessaries should be used as an alternative to surgery in younger women who still have plans for more deliveries and those engaging in physically strenuous work in order to reduce the risk of recurrence. Furthermore, in resource-limited settings like ours, where long follow-up periods are difficult and costly, younger women and those who suffer from postoperative vaginal cuff infection should be the priority in these follow-ups.
